# ^18^F-FDG and ^11^C-choline uptake in proliferating tumor cells is dependent on the cell cycle in vitro

**DOI:** 10.1007/s12149-018-01325-6

**Published:** 2018-12-26

**Authors:** Motoi Roppongi, Mitsuru Izumisawa, Kazunori Terasaki, Yasushi Muraki, Masanori Shozushima

**Affiliations:** 1 Division of Dental Radiology, School of Dentistry, Iwate Medical University, 1-3-27 Chuodori, Morioka, Iwate 020-8505 Japan; 2 Cyclotron Research Center, Iwate Medical University, 348-58 Tomegamori, Takizawa, Iwate 020-0603 Japan; 3 Division of Infectious Diseases and Immunology, Department of Microbiology, School of Medicine, Iwate Medical University, 2-1-1 Nishitokuta, Yahaba, Iwate 028-3694 Japan

**Keywords:** ^18^F-FDG, ^11^C-choline, Cell cycle

## Abstract

**Objective:**

Among different PET tracers, ^18^F-fludeoxyglucose (FDG) and ^11^C-choline are known to have a high tumor uptake correlated with a high mitotic index of tumor cells. Thus, the uptake of ^18^F-FDG and ^11^C-choline may be dependent on the cell cycle. In the present study, we examined the uptake of ^18^F-FDG and ^11^C-choline in cancer cell lines by cell cycle synchronization to clarify the biological properties of cancer cells with respect to each tracer.

**Methods:**

HeLa S3 Cells were synchronized by the double thymidine (TdR) block methods. ^18^F-FDG and ^11^C-choline were administered to synchronized cells, and the radioactivity per cell was measured to compare the cellular uptake of the tracers during S, G2/M, and G1 phases. Flow cytometry (FCM) was performed to measure the proportion of cells in G1, S, and G2/M phases. Furthermore, the levels of glucose transporter 1 (GLUT1) and choline transporter-like protein 1 (CTL1) in the cell were evaluated by FCM.

**Results:**

The uptake of ^18^F-FDG was the highest in S to G2/M phases, and markedly decreased in G1 phase. The uptake of ^11^C-choline reached its peak in G2/M, and decreased in G1 phase. The level of GLUT1 expression was similar to that of ^18^F-FDG uptake during the cell cycle, and the level of CTL1 expression was similar to that of ^11^C-choline uptake throughout the cell cycle.

**Conclusions:**

In this in vitro study, we demonstrated that ^18^F-FDG and ^11^C-choline had the highest uptake in S to G2/M phases and in G2/M phase, respectively, with a rapid decrease in G1 phase. These findings suggest that ^18^F-FDG and ^11^C-choline have a high accumulation in tumor cells with a high mitotic index. Furthermore, our study suggests that the expression of GLUT1 and CTL1 has cell cycle dependence, and the changes of ^18^F-FDG and ^11^C-choline accumulation seem to be caused by the above properties of these transporters.

## Introduction

PET is a diagnostic imaging modality in nuclear medicine that utilizes radioactive tracers with specific uptake in cancer cells. ^18^F-fluorodeoxyglucose (FDG) is the most common tracer and detects increased glucose metabolism in cancer cells [1]. ^11^C-choline is another tracer that detects phospholipid metabolism in the cell. Unlike ^18^F-FDG, ^11^C-choline does not accumulate in the brain under normal physiological conditions and is rarely excreted in urine during PET scanning; thus, it is used in the detection of brain tumors, as well as for pelvic masses such as prostate tumors [2]. Uptake of a PET tracer is an important indicator used to evaluate the treatment response and to predict disease outcomes. Halfpenny et al. [3] had found that head and neck cancer patients with ^18^F-FDG SUV (standardized uptake value) ≥ 10 in the primary tumor had a significantly lower survival rate. However, the uptake of ^18^F-FDG and ^11^C-choline to tumor tissue varies significantly within the same mass with identical tumor tissue and rate of progression. A study by Minn et al. [4] revealed ^18^F-FDG uptake to be higher in proliferating cells, suggesting that ^18^F-FDG uptake is dependent on the cell cycle. In cells, choline is used to synthesize phosphatidyl choline, which is an important constituent of the cellular membrane. Hara et al. [5] reported that the uptake of ^11^C-choline in cancer cells is proportional to the rate of the cell cycle, such that malignant tumors with a high frequency of cell division have a higher level of uptake. In addition, Glunde et al. [6] demonstrated a strong correlation between the level of abnormal choline metabolism in cancer cells and disease progression over time. These findings suggest that, as with the uptake of ^18^F-FDG, that of ^11^C-choline is also dependent on the cell cycle. Previously, some studies investigated associations between the uptake of ^18^F-FDG and cell cycle, such that the high expression of Ki 67 is labelled S phase [7, 8]. However, the relationship between tumor uptake and cell cycle other than S phase is still unknown. Furthermore, there are few reports about the relations between ^11^C-choline and cell cycle. In the present study, we sought to clarify the biological properties of cancer cells to interpret PET images in an appropriate manner. Specifically, we continuously changed the cell cycle in a human cancer cell line, HeLa S3, to examine changes in the uptake of ^18^F-FDG and ^11^C-choline over time.

## Materials and methods

### Cancer cell line and synchronization

Human cervical cancer cells HeLa S3 (RCB0191, Riken, Tsukuba, Japan) were cultured in minimal essential medium l-glutamine (MEM) medium (Thermo Fisher Scientific, Tokyo, Japan) supplemented with 10% fetal bovine serum (FBS; Invitrogen, Tokyo, Japan) in 5% CO_2_ at 37 °C. For cell culture, 50-mL flasks with a 25-cm^2^ surface area (Thermo Fisher Scientific, Tokyo, Japan) were used. Cells were cultured to a total number of 1–2 × 10^6^ cells per flask after synchronization. According to the protocol described previously by Knehr et al. [9], cells were synchronized by double thymidine (TdR) blocking using a high concentration of TdR (Sigma-Aldrich Co., St. Louis, USA). Specifically, cells were cultured in medium supplemented with 2 mM TdR for 24 h, and switched to TdR-free medium for 11 h. Cells were then cultured again in medium supplemented with 2 mM TdR for 14 h and switched to TdR-free medium for synchronization. Time 0 was defined as the end of synchronization, and flasks containing cells were rapidly cooled to 4 °C at 0, 2, 4, 5, 6, 7, 8, 10, and 11 h, and then stored at 4 °C.

### PET tracer injection and measurement of radioactivity

Two cancer-specific tracers of ^18^F-FDG and ^11^C-choline were used. All tracers were synthesized at the Nishina Memorial Cyclotron Center. ^18^F-FDG was synthesized using H_2_^18^O water in the first step, followed by the ^18^O(p, n)^18^F reaction. ^11^C-choline was synthesized according to the protocol described by Pascali et al. [10], using the on-column ^11^C-methylation technique. Specifically, ^11^C-CO_2_ was synthesized via a nuclear reaction with ^14^N(p, α)^11^C, followed by synthesis of ^11^C-methyliodid. For each tracer, a total of 370 kBq (10 µCi) per 1 mL culture medium was added to cells synchronized at each phase of the cell cycle. Cells were cultured with each tracer for 30 min at 37 °C and were quenched it to 4 °C and stopped metabolism. Thereafter, cells were then collected by trypsinization and washed three times with 4 °C phosphate buffer saline (PBS) supplemented with 5% FBS. From the preliminary experiment, the count level of both tracers confirmed that enough values were produced by 15 min. The incubation time was adopted to be 30 min which was twice as much as 15 min to ensure the stability of data. Radioactivity could measure enough value if it was over culture of 30 min and three times of washout. Radioactivity for each tracer in the cells was measured using a Gamma counter (Auto Well Gamma System ARC-2000, Aloka, Tokyo, Japan) and corrected for the half-life of each respective tracer. The number of cells was measured by an automated cell counter (TC20, Bio-Rad, Tokyo, Japan) to calculate the radioactivity per cell.

### Analysis by flow cytometry

The relative amount of DNA was measured using flow cytometry (FCM) (FACSCalibur, BD, San Jose, USA) to confirm synchronization of the cells. Specifically, cells were suspended in 2% Triton X-100 (Nacalai Tesque, Kyoto, Japan) to yield protoplasts and RNase (Sigma-Aldrich, Tokyo, Japan) was added to a final concentration of 0.5% before staining DNA with propidium iodide (PI) (Sigma-Aldrich, Tokyo, Japan) at a final concentration of 50 µg/mL. The cell suspension was prepared with 1 × 10^6^ cells/mL to measure the amount of nuclear DNA with FCM. The proportion of cells in G1, S, and G2/M phases was measured using ModFit LT 2.0 (Verity Software House, Top slam, ME, USA). The levels of glucose transporter (GLUT) and choline transporter-like protein (CTL) expression in the cell were measured by FCM using fluorescent antibodies for each protein. Specifically, GLUT was detected using anti-GLUT1-FITC antibody (R&D systems, Minneapolis, MN, USA) and CTL was detected using anti-CD92-FITC antibody (GeneTex, Ivine, CA, USA). Cells were collected by trypsinization, washed with PBS containing 3% FBS, and transferred to FACS tubes (5 mL round-button polystyrene tubes, Corning, NY, USA). Cells were then incubated in the dark for 30 min at room temperature with 10 µL of anti-GLUT1-FITC per 0.5 × 10^6^ cells/100 µL, and with 10 µL of anti-CD92-FITC antibody per 0.5 × 10^6^ cells/50 µL to detect GLUT1 and CTL1, respectively. Cells were then centrifuged twice with PBS containing 3% FBS for washing, and the concentration was adjusted to 0.5 × 10^6^ cells/400 µL for FCM to evaluate the expression of GLUT1 and CTL1. A total of 10,000 cells was counted for all FCM analyses.

### Data analysis

Statistical analyses were performed with InStat (GraphPad Software, San Diego, CA, USA). One-way ANOVA and post hoc test (Tukey multiple comparison test) were used to calculate significant differences among treatment groups. Means are separated in the bar chart, with T-bars indicating standard deviation. A *p* value of < 0.05 was regarded as significant.

## Results

### Cell cycle and FCM

**Fig. 1:**
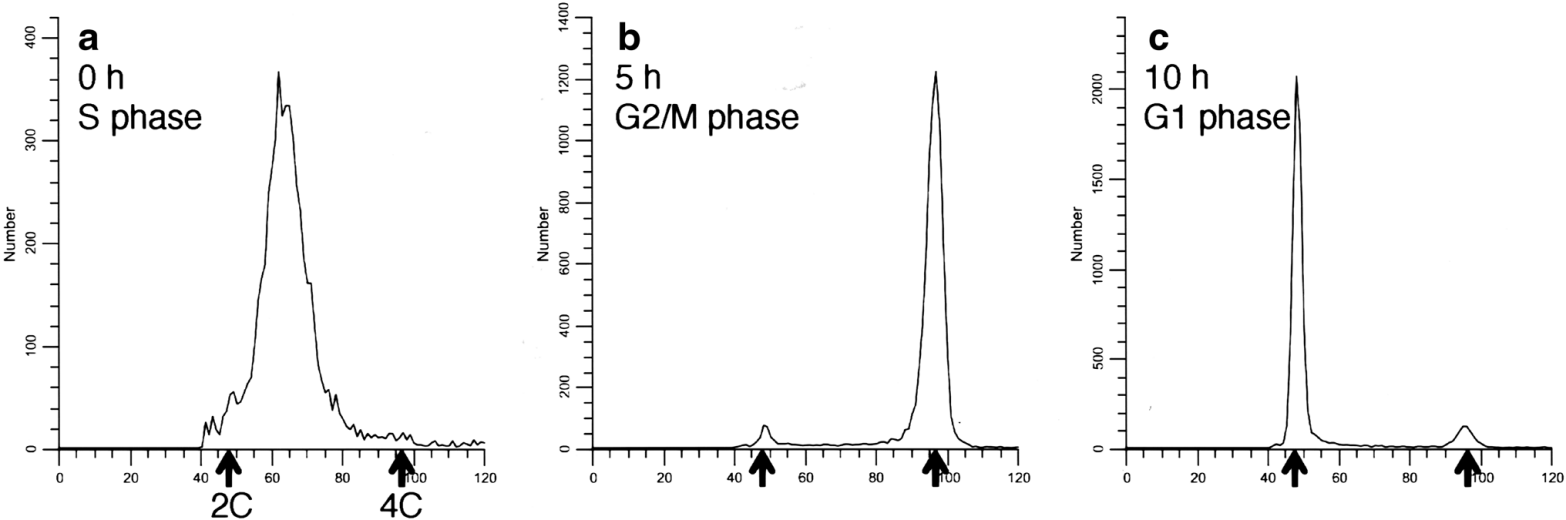
Flow cytometric analyses of HeLa S3 cells following synchronization. HeLa S3 underwent double TdR block, and the time from TdR-free medium for 0 (**a**), 5 (**b**), or 10 h (**c**). Cellular DNA was stained with PI and a flow cytogram was obtained. 2C and 4C in the *x*-axis indicate cells in G1 and G2/M phases, respectively

Cell synchronization with double TdR blocking was confirmed by FCM, and the correlation between the time from synchronization and the phases of cell cycle was examined. The results of FCM for DNA staining with PI are shown in Fig. 1, with the *x*-axis indicating the amount of DNA and the *y*-axis indicating the number of cells. At time 0 (immediately after synchronization), the peak of the histogram slightly shifted to the right compared with that in 2C, indicating synchronization in early S phase. After 5 and 10 h, the peak shifted from 4C (G2/M phase) to 2C (G1 phase). The data from FCM was analyzed using ModFit LT 2.0, and the proportion of cells in G1, S, and G2/M phases from the time cells were switched to TdR-free medium was measured in each sample (Table 1). The majority of cells were in S phase immediately after switching to TdR-free medium (99.7%), G2/M phase 5 h later (82.3%), and G1 phase 10 h later (77.6%).

**Table 1:** Percentage of cells enriched in specific cell cycle phases

The time from synchronization (h)	G1 (%)	S	G2/M
0	0.1	**99.7**	0.2
2	3.9	82.7	13.4
4	7.8	43.5	48.7
5	9.9	7.7	**82.3**
6	21.4	7.7	70.9
7	47.4	8.1	44.5
8	76.3	9.3	14.4
10	**77.6**	18.3	4.1
11	57.5	38.5	4.0

Data obtained by FCM was analyzed using ModFit LT 2.0 to calculate the proportion of cells in G1, S, and G2/M phases in each sample with respect to time after switching to TdR-free medium. Values indicating a high proportion of synchronized cells are shown in bold (average, *n* = 5)

### PET tracer uptake and cell cycle

**Fig. 2:**
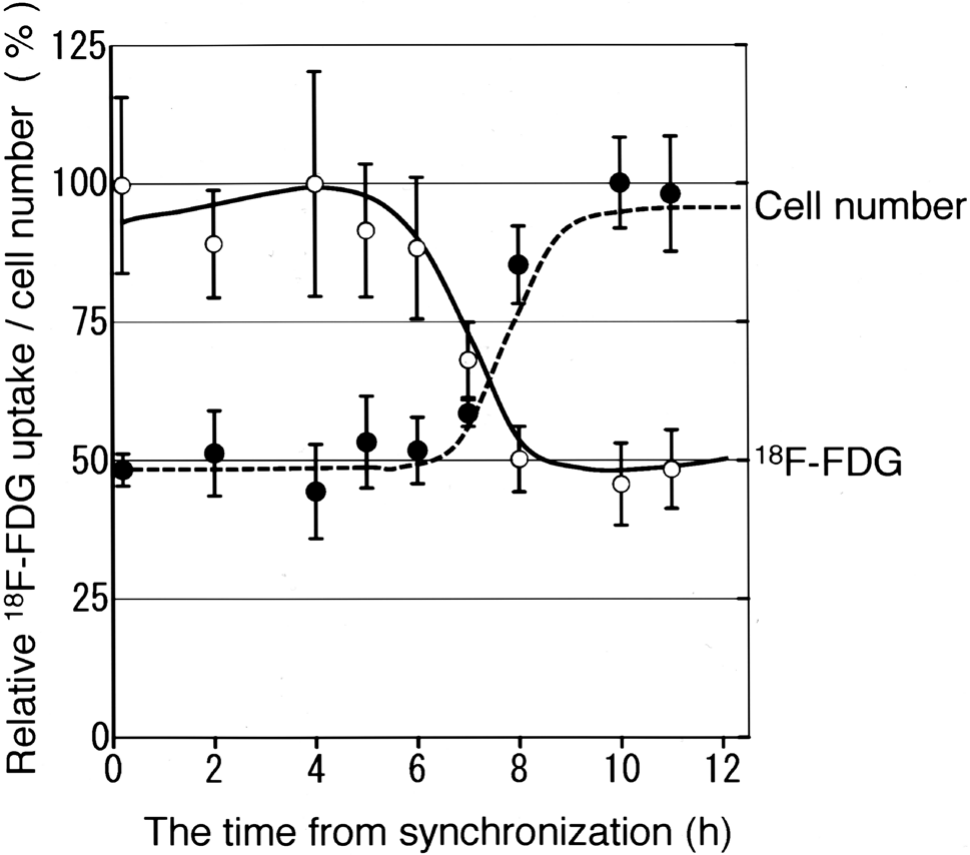
^18^F -FDG uptake and cell numbers after synchronization of HeLa S3 cells. ^18^F-FDG uptake and change in cell numbers based on the time from synchronization. The *x*-axis indicates the time from synchronization, and the *y*-axis indicates ^18^F-FDG uptake (open circle) and the number of cells (filled circle) expressed relative to the maximum (100%) (average, *n* = 5)

**Fig. 3:**
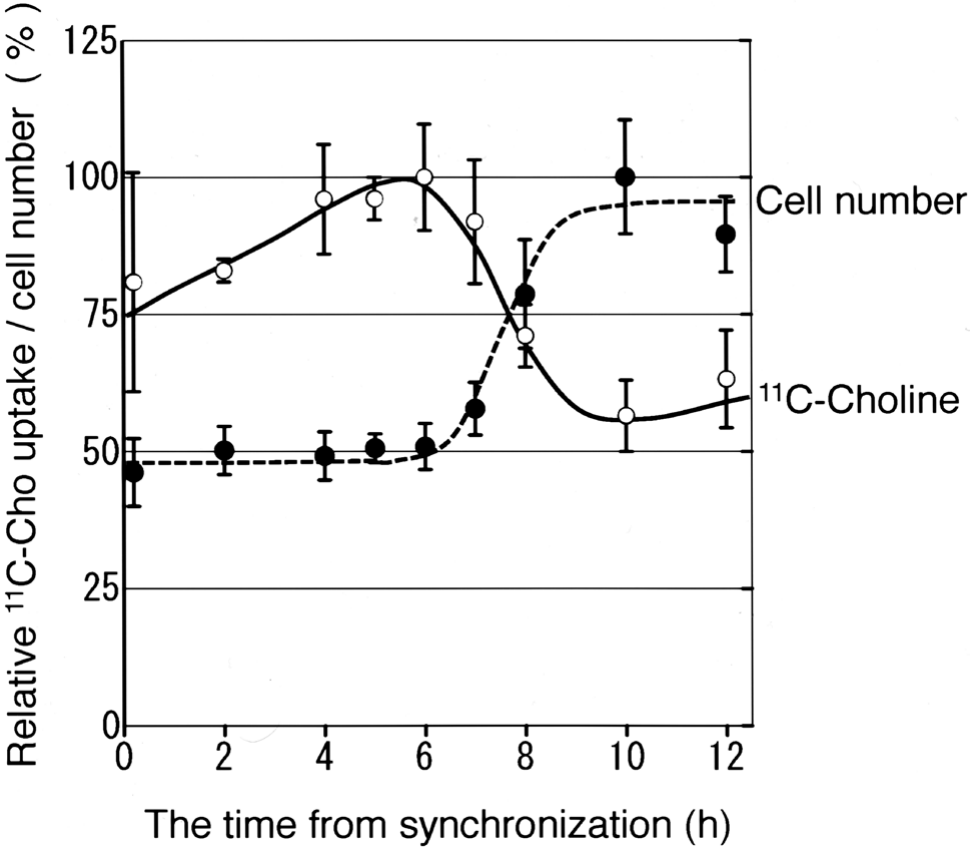
^11^C-choline uptake and cell numbers after synchronization of HeLa S3 cells. ^11^C-choline uptake and change in cell numbers based on the time from synchronization. The *x*-axis indicates the time from synchronization, and the *y*-axis indicates ^11^C-choline uptake (open circle) and the number of cells (filled circle) expressed relative to the maximum (100%) (average, *n* = 5)

The cellular uptake of ^18^F-FDG and ^11^C-choline, as well as changes in cell numbers in each phase of the cell cycle, is shown in Figs. 2 and 3. The *x*-axis indicates the time from synchronization, and the *y*-axis indicates ^18^F-FDG or ^11^C-choline uptake and the number of cells, which were expressed relative to the maximum level (100%). Based on the data presented in Table 1 and the overall change in the number of cells, the cells were in S phase 4 h after switching to TdR-free medium and were in G2/M phase until 7 h after switching to TdR-free medium before entering G1, where the number of cells almost doubled. The uptake of ^18^F-FDG (Fig. 2) reached its maximum immediately after synchronization and at 4 h after synchronization in S phase and decreased gradually to approximately 50% of the maximum amount by 10 h after synchronization in G1. The uptake of ^11^C-choline (Fig. 3) increased in S phase and reached its maximum 5–6 h after synchronization in G2/M, and decreased to approximately 60% of the maximum amount by 10 h after synchronization in G1.

### GLUT1 and CTL1 expression and the cell cycle

**Fig. 4:**
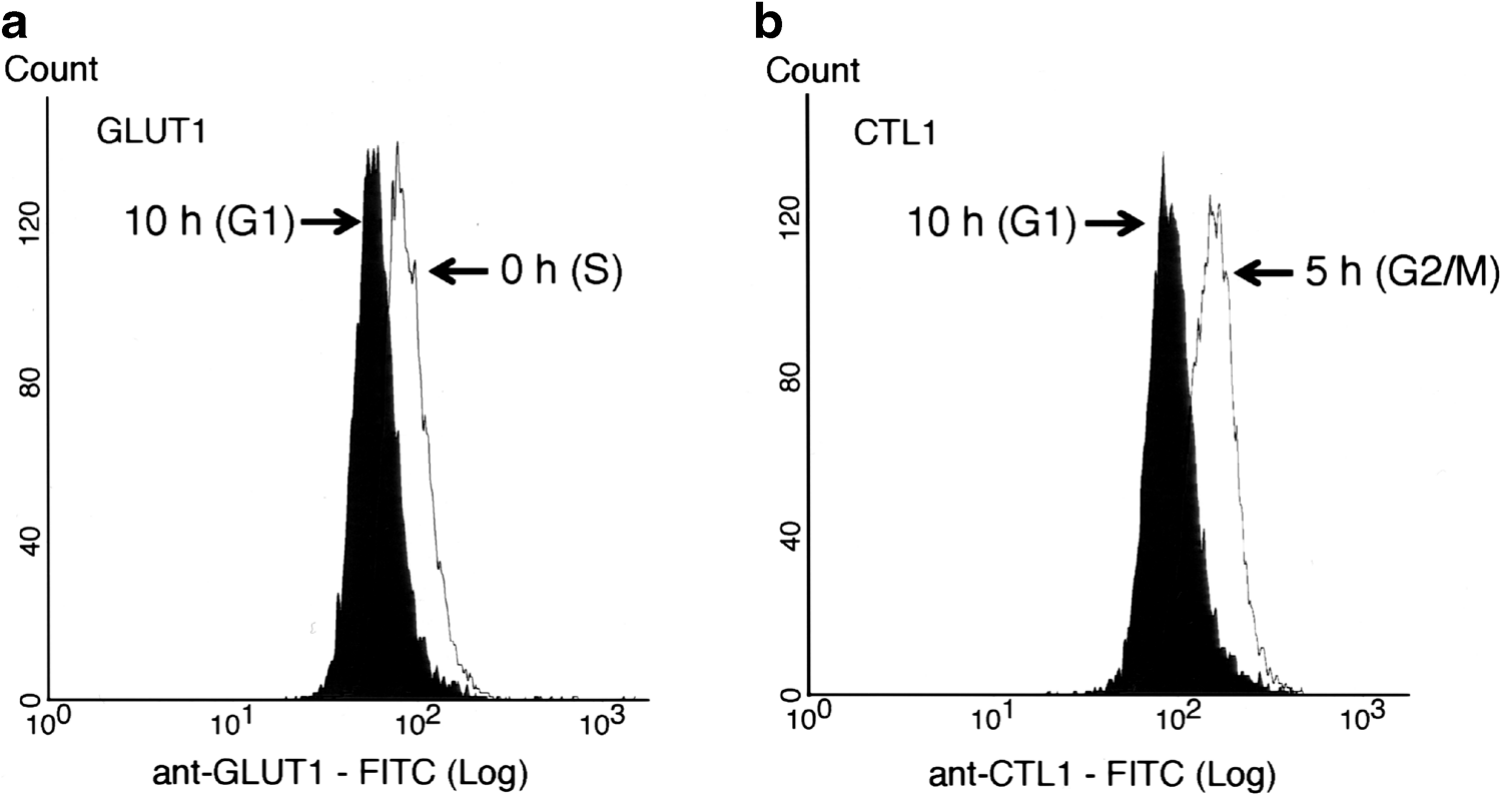
Flow cytograms of the expression of GLUT1 and CTL1. Flow cytogram with fluorescence labeling using anti-GLUT1-FITC (**a**) and anti-CTL1-FITC (**b**)

**Fig. 5:**
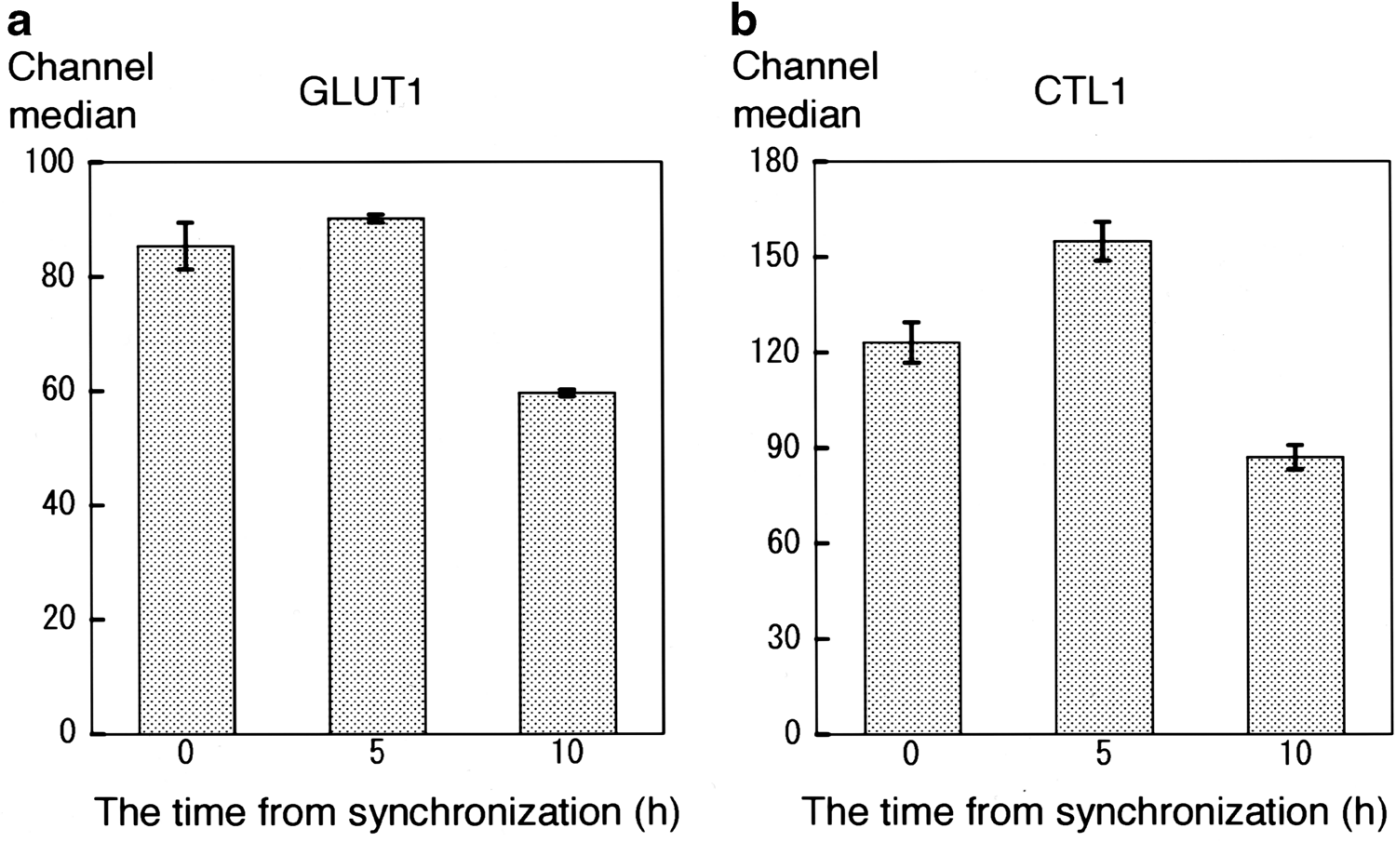
Flow cytometric analyses of the expression of GLUT1 and CTL1. The fluorescence intensity of anti-GLUT1-FITC (**a**) and anti-CTL1-FITC (**b**) was measured as the channel median. The *x*-axis indicates the time after switching to TdR-free medium. The Tukey multiple comparison test was performed to compare GLUT1 and CTL1 expression over time. The level of GLUT1 expression was significantly different between 0 and 5 h (*p* < 0.05), 0 and 10 h (*p* < 0.001), and 5 and 10 h (*p* < 0.001). The level of CTL1 expression was significantly different across all time points (*p* < 0.001) (average, *n* = 5)

The expression of GLUT1 and CTL1 with respect to the cell cycle was compared by FCM. The histograms for anti-GLUT1-FITC and anti-CTL1-FITC antibodies are shown in Fig. 4, with the *x*-axis indicating the fluorescence intensity and the *y*-axis indicating the number of cells. The peak in GLUT1 expression shifted to the left from S phase (0 h) to G1 phase (10 h), and the fluorescence intensity of GLUT1 decreased with time. Similarly, CTL1 expression peaked in G2/M (5 h) and shifted to the left after G1 (10 h) with decreasing fluorescence intensity. Furthermore, fluorescence intensity for cells in S, G2/M, and G1 phases was quantified as the median channel value (ch. median) (Fig. 5). The level of GLUT1 expression was high in S phase (0 h), reached its maximum in G2/M phase (5 h), and decreased to its minimum in G1 phase (10 h). The level of CTL1 reached its maximum at 5 h and decreased at 10 h. Based on the Tukey multiple comparison test, there were significant differences between the levels of GLUT1 expression at 0 and 5 h (*p* < 0.05), and among the levels of CTL1 across all time points (*p* < 0.001).

## Discussion

In this in vitro study, we showed that ^18^F-FDG and ^11^C-choline had the highest uptake in S to G2/M phases and in G2/M phase, respectively, with a rapid decrease in G1 phase. Furthermore, the expression of GLUT1 and CTL1 has cell cycle dependence, the level of GLUT1 expression was similar to change of ^18^F-FDG uptake during the cell cycle, and the level of CTL1 expression was similar to that of ^11^C-choline uptake throughout the cell cycle.

The majority of normal human cells at any one point are in the resting phase (G0 phase) of the cell cycle where they do not replicate. Cells exit G0 upon external growth stimulation, and cycle through G1 (Gap 1), S phase (DNA synthesis), G2 (Gap 2), and M phase (mitotic phase) before returning to G1. The cell cycle of HeLa S3 cells used in this study is well-characterized, with S phase lasting 9 h and G2/M phase lasting 3 h [11]. Several methods have been developed to synchronize cells, including S phase cell cycle arrest with TdR, G1 phase cell cycle arrest with hydroxyurea [12], and harvesting cells in M phase by shaking, because cells in M phase have low adherence to the culture surface. In the present study, we chose the double TdR block method described previously by Knehr et al. [9], as it is reversible and produces a high yield of synchronous cells. This method is based on the high concentration of TdR inducing cell cycle arrest at S phase for cells that were already in S phase, and entrance into G1 for cells that were in other phases of the cell cycle. The time from the first TdR block and the second TdR block is critical for obtaining homogenously synchronized cells; thus, we monitored flow cytograms to determine the time between blocking. Using ModFit to calculate the proportion of synchronized cells, we demonstrated synchronization to be successful, as 99.7% of cells were in S phase immediately after switching to TdR-free medium, 82.3% were in G2/M after 5 h, and 77.6% were in G1 after 10 h. The observed gradual decrease in synchronization over time may be because of the heterogeneity of HeLa S3 cells.

Several studies have reported the correlation between tumor cell proliferation and uptake of ^18^F-FDG [13–15]. These studies collectively demonstrated that ^18^F-FDG uptake is higher in cells with a rapid proliferation rate than in those with a slower proliferation rate, suggesting it to be an indicator of malignancy. Our findings further suggest that this observation is because of the high proportion of cells in S and G2/M phase in tumors. In a study of ^18^F-FDG PET, Minn et al. [4] revealed that the uptake of ^18^F-FDG does not depend on the histological grade of head and neck cancer and is instead dependent on the proliferative capacity of tumors. Therefore, tissues with a high proportion of cancer cells in S and G2/M phases have a higher uptake of ^18^F-FDG. These findings suggest that ^18^F-FDG uptake in cells in G0 and G1 is relatively low. In the present study, we examined the uptake of ^18^F-FDG per cell by synchronizing cells from S to G1 phase. We demonstrated that ^18^F-FDG uptake was high in S and G2/M phases, but rapidly decreased in G1. This in vitro finding supports the results by Minn et al.

In addition to tumor cells, ^18^F-FDG accumulates in highly proliferating cells. Sugawara et al. [16] reported that the administration of granulocyte colony-stimulating factor (G-CSF) in patients with a low neutrophil count resulting from chemotherapy or radiation therapy activated neutrophil progenitors in bone marrow, leading to an increased uptake of ^18^F-FDG in the bone marrow. Furthermore, the activation of murine T cells by lectin was found to cause the rapid uptake of ^3^H-TdR, a marker of S phase, as well as of ^3^H-DG (^3^H-deoxyglucose) [17]. Consistent with our findings, these studies suggest that the uptake of ^18^F-FDG is not limited to cancer cells, because it also occurs in proliferating cells such as those of the immune system. Cancer cells undergoing rapid proliferation utilize glucose as the source of metabolism. Glucose is transported into the cells via GLUTs, which change their structure upon binding to specific sites of GLUT family members expressed in the cell [18, 19].

Among the 13 GLUTs identified to date, GLUT1, GLUT3, and GLUT4 have a high affinity to glucose. The expression of GLUTs is dependent on hypoxia-inducible factor, growth factor, and several oncogenes [20]. In particular, GLUT1 expression has been identified in many cancer cells [20]. GLUT1 expression is also associated with tumor aggressiveness. High levels of glycolysis and increased expression of GLUT1 have been identified in advanced cancer stages, and are associated with poorer treatment outcomes [21, 22]. As with glucose, ^18^F-FDG enters cells via GLUT, and is phosphorylated by hexokinase and ATP. However, it is not metabolized and is trapped in cells. Although there is a strong correlation between the uptake of ^18^F-FDG and GLUT expression, no correlation was found between the uptake of ^18^F-FDG and hexokinase expression in human cancer cells [23, 24]. In the present study, we examined the association between the cell cycle and GLUT1 expression and demonstrated that GLUT1 expression increased in S and G2/M phases and decreased in G1. As this pattern was consistent with the uptake of ^18^F-FDG, the cell cycle dependence of ^18^F-FDG uptake may reflect the cell cycle dependence of GLUT1 expression.

Choline is one biological factor that plays essential roles in numerous cells. Specifically, it is required for the synthesis of phosphatidylcholine and sphingomyelin, which constitute the cellular membrane. hCTL1c (CTL1) is identical to human CD antigen (CD92), and is responsible for the transportation of choline required in all cells. Currently, PET/CT with ^11^C-choline and ^18^F-choline is performed clinically for the detection of head and neck, breast, prostate, colorectal, and lung cancers [25–27]. A study using the colorectal cancer cell line HT-29 demonstrated that choline was transported via CTL1 [28]. In addition, high expression of CTL1 was observed in lung and esophageal cancer cells [29, 30]. In the present study, the uptake of ^11^C-choline increased in S phase and reached its maximum in G2/M before rapidly decreasing in G1. As the level of CTL1 (CD92) expression was similar according to FCM, our findings suggest that the expression of CTL1 is dependent on the cell cycle and that ^11^C-choline is transported into cells via CTL1. Cells in G2/M increase in size as they prepare for cell division; therefore, there may be an increased need for choline to synthesize the cell membrane.

In addition to the localization of PET tracers, accumulation amount of the radiopharmaceutical is also an important indicator of cancer when using PET. A high uptake of ^18^F-FDG with a high mitotic index and high density of tumor cells has been reported [4, 31]. In addition to these factors associated with tracer uptake, our in vitro study demonstrated that the high uptake of ^18^F-FDG and ^11^C-choline reflects the cell cycle dependence of tracer uptake.

## Conclusions

We synchronized HeLa S3 cells in S, G2/M, and G1 phases of the cell cycle, and revealed that uptake of ^18^F-FDG and ^11^C-choline was high in S and G2/M phases, and G2/M phase, respectively, and that the uptake of both tracers decreased rapidly in G1. Furthermore, the uptake of these tracers was dependent on the cell cycle, which may be due to the cell cycle-dependent expression of GLUT1 and CTL1 expressed in HeLa S3 cells.
